# Technogenic Fiber Wastes for Optimizing Concrete

**DOI:** 10.3390/ma15145058

**Published:** 2022-07-20

**Authors:** Sergey Klyuev, Roman Fediuk, Marina Ageeva, Ekaterina Fomina, Alexander Klyuev, Elena Shorstova, Linar Sabitov, Oleg Radaykin, Sergey Anciferov, Diana Kikalishvili, Afonso R. G. de Azevedo, Nikolai Ivanovich Vatin, Mugahed Amran

**Affiliations:** 1 Belgorod State Technological University Named after V.G. Shukhov, 308012 Belgorod, Russia; ageevams@yandex.ru (M.A.); fomina.katerina@mail.ru (E.F.); kuzik_alena@mail.ru (E.S.); anciferov.sergey@gmail.com (S.A.); di_ki93@mail.ru (D.K.); 2 Polytechnical Institute, Far Eastern Federal University, 690922 Vladivostok, Russia; 3 Peter the Great St. Petersburg Polytechnic University, 195251 St. Petersburg, Russia; vatin@mail.ru; 4 Kazan Federal University, 420008 Kazan, Russia; sabitov-kgasu@mail.ru (L.S.); olegxxii@mail.ru (O.R.); 5 Kazan State Power Engineering University, 420066 Kazan, Russia; 6 LECIV—Civil Engineering Laboratory, UENF—State University of the Northern Rio de Janeiro, Av. Alberto Lamego, 2000, Campos dos Goytacazes, Rio de Janeiro 28013-602, RJ, Brazil; afonso.garcez91@gmail.com; 7 Department of Civil Engineering, College of Engineering, Prince Sattam Bin Abdulaziz University, Alkharj 16273, Saudi Arabia; m.amran@psau.edu.sa; 8 Department of Civil Engineering, Faculty of Engineering and IT, Amran University, Amran 9677, Yemen

**Keywords:** fiber concrete, cement composite, strength, waste utilization

## Abstract

A promising method of obtaining mineral fiber fillers for dry building mixtures is the processing of waste that comes from the production of technogenic fibrous materials (TFM). The novelty of the work lies in the fact that, for the first time, basalt production wastes were studied not only as reinforcing components, but also as binder ones involved in concrete structure formation. The purpose of the article is to study the physical and mechanical properties of waste technogenic fibrous materials as additives for optimizing the composition of raw concrete mixes. To assess the possibility of using wastes from the complex processing of TFM that were ground for 5 and 10 min as an active mineral additive to concrete, their chemical, mineralogical, and granulometric compositions, as well as the microstructure and physical and mechanical characteristics of the created concretes, were studied. It is established that the grinding of TFM for 10 min leads to the grinding of not only fibers, but also pellets, the fragments of which are noticeable in the total mass of the substance. The presence of quartz in the amorphous phase of TFM makes it possible to synthesize low-basic calcium silicate hydrates in a targeted manner. At 90 days age, at 10–20% of the content of TFM, the strength indicators increase (above 40 MPa), and at 30% of the additive content, they approach the values of the control composition without additives (above 35 MPa). For all ages, the ratio of flexural and compressive strengths is at the level of 0.2, which characterizes a high reinforcing effect. Analysis of the results suggests the possibility of using waste milled for 10 min as an active mineral additive, as well as to give better formability to the mixture and its micro-reinforcement to obtain fiber-reinforced concrete.

## 1. Introduction

An important direction in the development of concrete science is to minimize the use of cement as a result of its partial replacement with various fillers, including those obtained from production waste [1,2,3]. Many production wastes are sufficiently studied for use as structure-forming components of a binder and reinforcing elements. Examples from recent years include the study of nano-montmorillonite and carbon nanotubes [4], zeolite and metakaolin [5], recycled concrete aggregates [6], calcium silicate hydrates [7], etc.

A promising direction for obtaining mineral fiber fillers for dry building mixtures is the processing of waste from the production of technogenic fibrous materials (TFM) [4,5,6].

**Figure 1:**
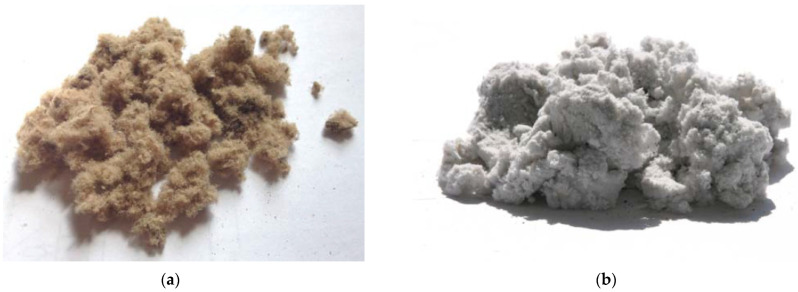
Appearance of finely divided technogenic fibrous materials obtained from: (**a**) corrugated cardboard, (**b**) coated writing paper.

The most studied type of TFM is pulp and paper waste, consisting mainly of fibers with a length of 0.1 to 5 mm, of plant origin, isolated from coniferous and hardwood (including technical pulp and waste paper), the stems and bast of annual plants, and seed pods and leaves of some plants or mineral (asbestos) origin (Figure 1) [7,8,9].

In the world, about 15 million tons of pulp and paper waste are generated annually, of which 12 million tons are suitable for processing [10,11,12,13].

Cellulose fiber has an amorphous structure, is insoluble in water, is resistant to acids and alkalis, and has a pH value in the range of 4 to 12 [14,15]. Cellulose fiber is insoluble in organic solvents (for example, oils), physiologically and toxicologically safe, and easy to use [16,17]. The use of natural cellulose fiber helps to improve the technological characteristics of mortar mixtures due to the fact that the fiber structure has a high absorbing and releasing ability in relation to water and organic liquids [18,19]. Due to the retention of moisture in the structure of cellulose microfibers, a concentrated aqueous medium is created between the mineral particles, which reduces the release of moisture into the hydrophilic base and eliminates the risk of shrinkage cracks. Since the fibers distribute and “transport” moisture from the lower layers of mortar mixtures to the upper ones, the drying of the upper layer is excluded [20,21]. At the same time, the maturation of the product occurs evenly [22,23].

**Figure 2:**
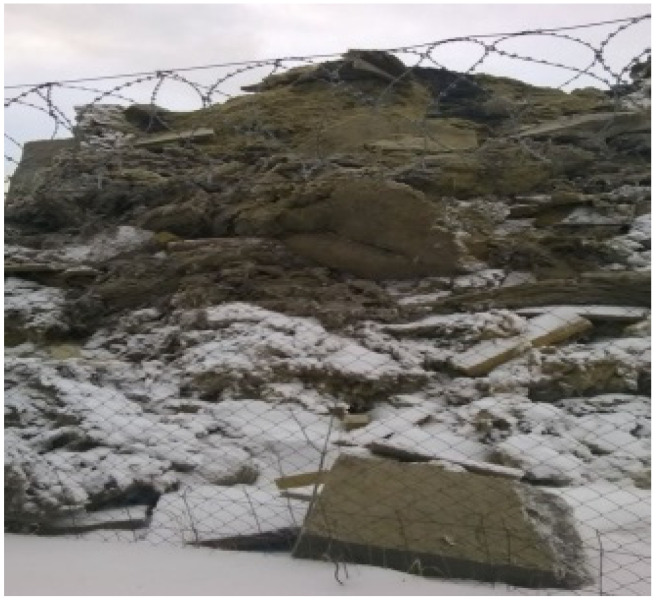
Waste production of basalt fiber insulation in a storage site.

A less studied type of TFM is the waste of mineral wool heat-insulating materials, for example, basalt fiber insulation [24,25]. With production volumes in Russia at the level of 50 thousand tons/year, up to 4 tons/year of waste is generated. Due to the relatively low bulk density (ρ = 200–220 kg/m^3^), storage areas occupy a large share of the area in factory warehouses and landfills (Figure 2).

One of the important tasks of processing secondary basalt fiber, in bulk or compacted states, is significantly reducing its volume, imparting properties of flowability and generating the possibility of obtaining commercial products, such fibers of various lengths for subsequent use in the construction industry [26,27].

It is advisable to use a step-by-step process for the complex processing of basalt fibrous waste:

− preliminary destruction of the original basalt fibrous waste, removal of non-fibrous inclusions [28];
− deagglomeration [29];
− classification with the removal of spillage and speck (“beadlet”) [30];
− providing various sizes of fibers for their use in composite mixtures for various technological purposes, incl. for 3D technologies in construction [31,32].

Obtaining agglomerated, highly concentrated microfiber fillers has a number of technological advantages: increased flowability and better transport ability, which ensures a more uniform distribution of fibers in macro and micro volumes of a composite mixture with heterogeneous components, and expands the scope of the use of basalt waste (fibers) in an agglomerated form for various technological purposes (in the form porous aggregates, thermal insulation, adsorbents, etc.) [33].

The effects of using technogenic fiber in mortar or concrete [33,34,35]:

− increase in the crack resistance of the product (resistance to shrinkage and operational cracks);
− imparting thixotropic properties of the mortars;
− improving the fixing ability on a vertical surface, preventing tiles from slipping (if in tile adhesive);
− reduction in shrinkage that occurs during the maturation of mortar or concrete;
− increase in frost-resistant properties of products;
− improving the rheological properties of the mortar and increasing the time of work with them.

The use of various industrial fibers for reinforcing concrete is sufficiently studied, for example, basalt and polypropylene [36], steel, glass, and carbon ones [37]. However, the use of recycled waste, not only as fiber, but also as an active additive in the cement system, is insufficiently studied.

The novelty of the work lies in the fact that, for the first time, basalt production wastes are studied not only as reinforcing components, but also as binder components, involved in concrete structure formation.

The purpose of the article is to study the physical and mechanical properties of waste technogenic fibrous materials for use as additives for optimizing the composition of raw concrete mixes.

## 2. Materials and Methods

### 2.1. Materials

Portland cement CEM I 42.5 N (Belgorod Cement, Belgorod, Russia) according to EN-197 was used as a binder. As an active mineral additive to concrete, wastes from the complex processing of TFM (mineral wool) were used. Microsilica, which is a technogenic waste from metallurgy, was used to provide the pozzolanic reaction.

Table 1 lists the chemical composition and specific surface area of the raw materials. In particular, glass-forming oxides SiO_2_, CaO, MgO, Al_2_O_3_, Fe_2_O_3_, Na_2_O, TiO_2_, and K_2_O impurities represent mineral wool. The amount of SiO_2_ is 44.11%, the specific surface area is 200 m^2^/kg, the bulk density is 1366 kg/m^3^; and true density is 2884 kg/m^3^.

The superplasticizer “PFM-NLK” (Poliplast, Moscow, Russia) was used in the mixes. For the developed mortars, sand with a particle size of 1.5–2.5 mm was used.

### 2.2. Mix Design

In the work, samples of 70 × 70 × 70 mm in size (according to Russian standard GOST 310) were molded with different waste content (10–40% by weight of cement) and the corresponding replacement of part of the cement. The water to cement ratio (W/C) was 0.4 and the sand/binder ratio was kept constant at 2.75. The compositions of concrete mixes are given in Table 2. Short-term mixing of the dry mixture with fiber for 60 s ensured the uniform distribution of basalt fiber.

All mixes were designed based on the conditions of equal workability, providing a slump of 19 cm and a slump flow of 43 cm.

### 2.3. Methods

To assess the possibility of using wastes ground for 5 and 10 min for the complex processing of TFM as an active mineral additive to concrete, their chemical and mineralogical compositions were studied using an X-ray fluorescence spectrometer. Using a scanning electron microscope, the shape and size of the particles were visually assessed before and after grinding.

The specific surface area was determined by two complementary methods using the PSH-2 device (Khodakov’s Devices, Moscow, Russia) and the Sorbi-M device (4-point BET method), (Sorbi, Moscow, Russia). The volume of pores with a radius <19.4 nm was also calculated.

The microstructure of the materials was studied using a Mira 3 scanning electron microscope (Teskan, Brno, Czech Republic).

Compressive and flexural strength tests were carried out after 7, 28 and 90 days according to the Russian standard GOST 310.

## 3. Results and Discussion

### 3.1. Comprehensive Study of the Technogenic Fibrous Material

**Figure 3:**
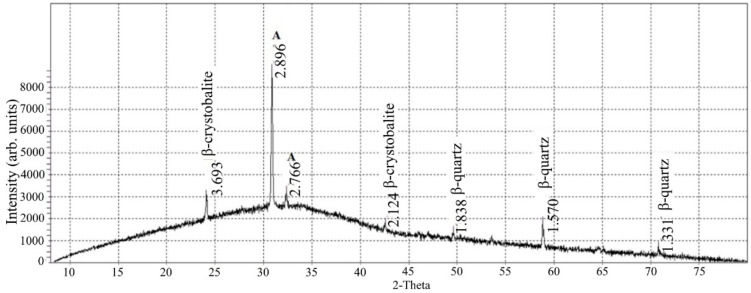
XRD pattern of waste basalt TFM: A—augite Ca(Mg,Fe)Si_2_O_6_ or anorthite CaAl_2_Si_2_O_8_.

Silica in this type of technogenic raw material is in an amorphous highly dispersed state, as evidenced by the maximum peak in the region of 31–33° (Figure 3).

The main crystalline phases of TFM that belong to plagioclases are augite Ca(Mg,Fe)Si_2_O_6_ and anorthite CaAl_2_Si_2_O_8_, which is characteristic of crystalline basalt. These phases begin to crystallize at temperatures above 900 °C during the melting of basalt in the production of mineral fiber.

The presence of crystalline phases of quartz in the β-modification is evidenced by characteristic diffraction reflections. There is a high-temperature polymorphic modification of quartz β-crystobalite. The presence of quartz in the amorphous phase causes high solubility in saturated alkaline solutions of the hydrated binder and active interaction with the Ca(OH)_2_ released as a result of the hydration of clinker minerals. These processes allow the targeted synthesis of low-basic calcium silicate hydrates during hydration, which are responsible for the strength properties of the finished building product.

**Figure 4:**
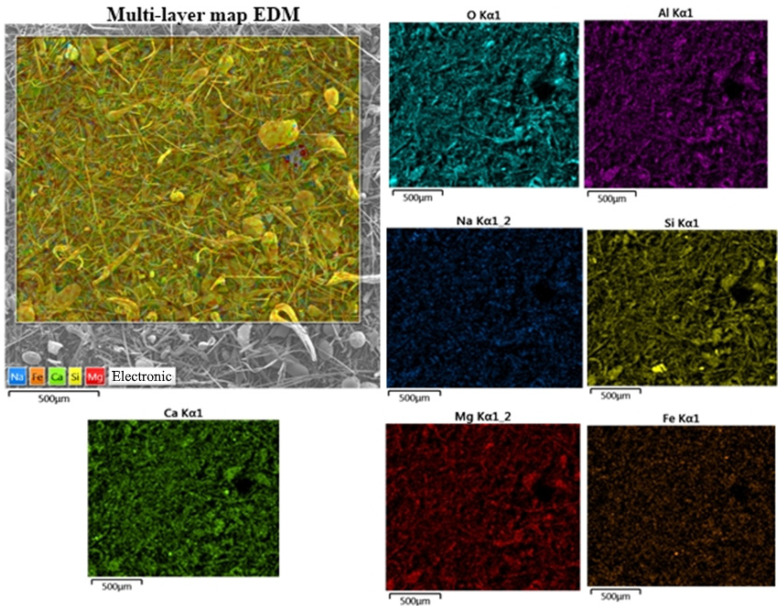
Distribution of chemical elements included in mineral wool waste over the surface.

The results obtained are confirmed by the energy dispersive microanalysis (EDM) of waste performed with the construction of a map of the distribution of chemical elements over the surface. Results also indicate the absence of additional inclusions in the material due to preliminary screening (Figure 4).

**Figure 5:**
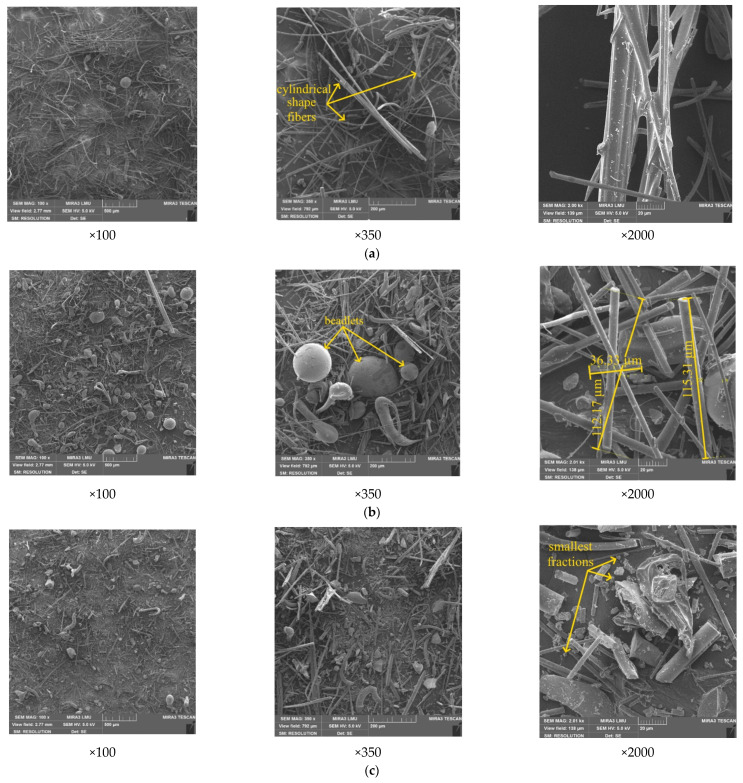
SEM images of mineral wool waste: (**a**) in its original form, (**b**) after grinding for 5 min, and (**c**) after grinding for 10 min.

SEM images obtained using a scanning electron microscope (Figure 5a) show that the mineral wool waste particles in their original form are represented by mainly cylindrically shaped fibers of various sizes and lengths. Due to the inhomogeneous properties of the melt, in the process of dispersion, along with the mineral fiber, so-called “beadlets” are formed from the solidified melt of spherical, droplet, and elongated shapes (Figure 5b). In addition, a large number of particles have various kinds of defects.

When grinding using a ball mill for 5 min, the size of the fibers is significantly reduced. First, there is the most intensive grinding of thin and long fibers; as a result, the ratio of length to diameter decreases. The beadlets themselves remain largely unchanged (Figure 5b), but sometimes their size exceeds the size of the fibers.

Further grinding for 10 min leads to grinding not only the fibers, but also the beadlets, the fragments of which are noticeable in the total mass of the substance. There is a large number of the smallest nanosized crushed fractions, however, there is also a significant amount of larger particles of a fibrous form (Figure 5c).

**Figure 6:**
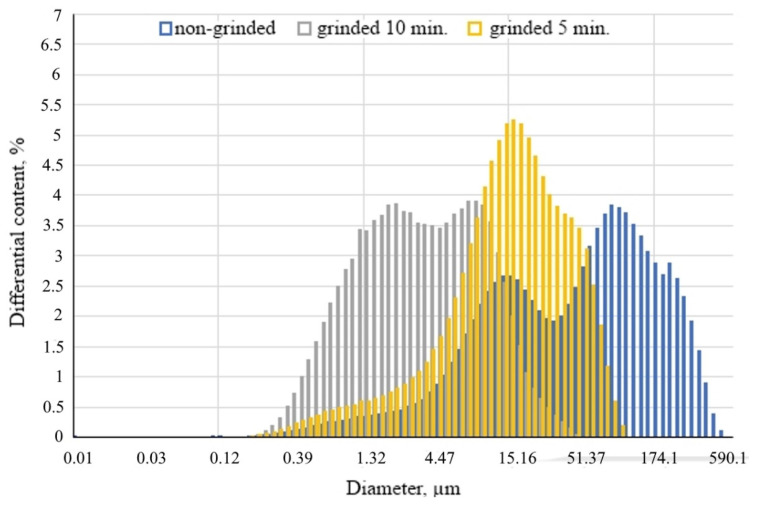
Particle size distribution of mineral wool production waste (TFM).

This is consistent with the obtained granulometric composition of the powders (Figure 6).

Figure 6 shows that during grinding there is a significant shift in the graphs to the area of the smallest particles with their smoother distribution over fractions, so the source material is characterized by a bimodal graph with a predominance of large particles (the average particle size is 91 μm (Table 3)). The particle size distribution curve after 5 min of grinding is characterized by one pronounced peak; the average particle size is 50.36 μm. The last graph is smoothly distributed in the region of small particles. The average diameter is about 14 µm, and 90% of the substance is less than 45 µm.

It should be noted that the finely ground TFM waste is characterized by a finely porous structure with a pore volume R < 19.4 nm of 0.023 cm^3^/g (Table 4).

**Figure 7:**
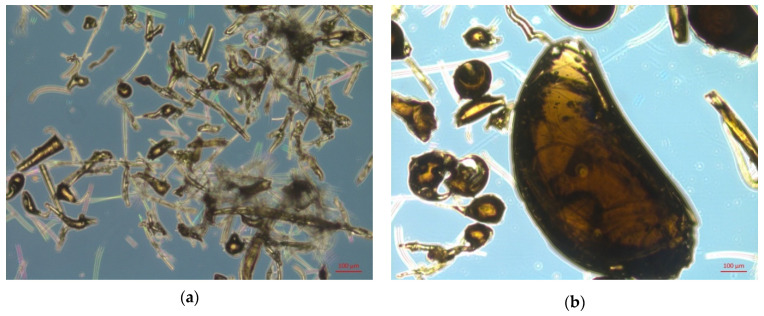
Basalt at a specific surface area of 500 m^2^/kg: (**a**) total TFM particles mass; (**b**) TFM beadlets.

According to microstructural analysis, porosity can be due to the presence of defects both inside and on the surface of fine particles (Figure 7a). There are various defects in the form of cracks and shells on the surface of the TFM beadlets (Figure 7b).

### 3.2. Properties of Concrete with TFM

The selected consumption of polycarboxylate superplasticizer with a high water-reducing capacity (40%) made it possible to maintain the slump within 20 cm for almost all compositions (Table 5).

Only at a dosage of TFM in the amount of 40 wt. % value of the slump decreased to 16 cm. At the same time, the parameter of the slump flow was kept at the level of 48–51 cm, allowing its decrease in the amount of TFM 30–40 wt. % up to 46 cm.

The density of the fresh mixture modified with FMT reduced when the dosage increasing. The decrease in the density of the hardened concrete samples is justified by the fact that the average density of cement hydration products is lower than the average density of the initial mixture.

**Figure 8:**
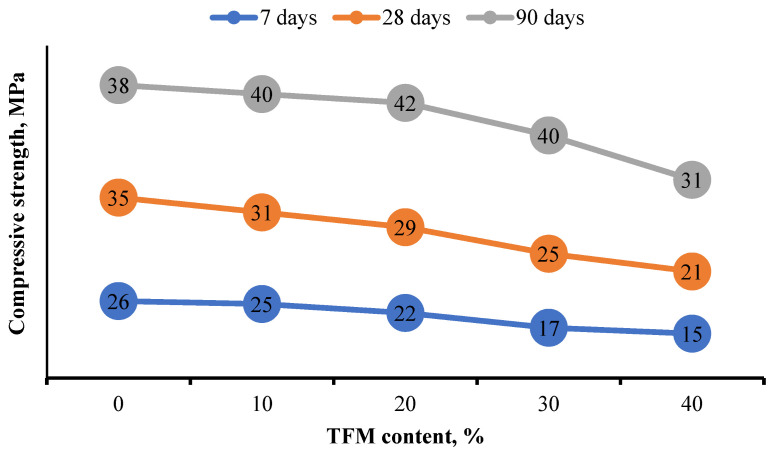
Dependence of concrete compressive strength on the consumption of additive TFM.

Compositions with 40% TFM in all periods of hardening have the lowest strengths, with a 50% drop compared to the control composition. For all the mixtures only at the age of 7 and 28 days, the compressive strength decreased (by about 20–40% compared to the control samples) as the content of mineral wool waste increased (Figure 8). Correlations between samples with different fiber additions were necessary to find the optimal content, which has a deep practical meaning.

However, at the age of 90 days, at 10–20% of the content of TFM, the strength indicators increase, and at 30% of the content of the additive they approach the values of the control composition without additives. The decrease in compressive strength of samples with the replacement of cement by 30 and 40% of TFM is explained by supersaturation and weakening of the hardening system, confirming the optimal content of this component at the level of 20%.

Good early compressive strength results (7 days) are noted; even at 40% replacement of cement by TFM, the early compressive strength reaches 15 MPa. This allows you to speed up the production of concreting. The increase in strength may be associated with the pozzolanic reaction, the completeness of which increases most at a later age [38,39,40,41,42,43,44,45,46,47,48,49,50,51,52,53,54]. In addition, the microsize of the grains of TFM waste used in this study increases the compressive strength of the mortar, acting as a compactor.

The decrease in strength at 28 days of age may be due to the high water demand of the finely ground filler, which intensively absorbs water, slowing down the rate of hydration. However, in the future, the filler can release stored water, providing long-term hydration. Based on the test results mentioned above, the inclusion of 10–20% mineral wool waste is optimal.

**Figure 9:**
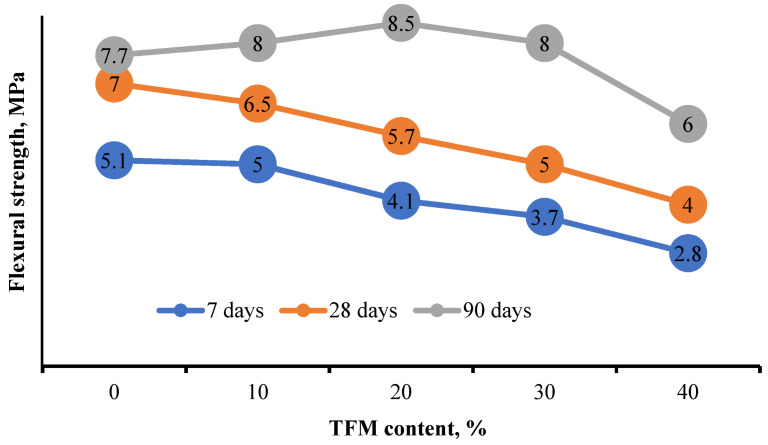
Dependence of concrete flexural strength on the consumption of additive TFM.

Good results were obtained in the study of flexural strength (Figure 9). The optimal content of TFM in the amount of 20% leads to the values of bending strength at the age of 7 days 4.1 MPa, at the age of 28 days 5.7 MPa, and at the age of 90 days 8.5 MPa. For all ages, the ratio of flexural and compressive strengths is at the level of 0.2, which characterizes a high reinforcing effect.

Thus, the analysis of the results suggests the possibility of using waste milled for 10 min as an active mineral additive, as well as to give better formability to the mixture and its micro-reinforcement to obtain fiber-reinforced concrete [55,56,57,58,59,60,61,62,63,64,65]. The presence of quartz in the amorphous phase causes high solubility in saturated alkaline solutions of the hydrated binder and active interaction with Ca(OH)_2_ released as a result of the hydration of clinker minerals.

## 4. Conclusions

The physico-mechanical properties of waste technogenic fibrous materials (TFM) used as additives for optimizing the composition of raw concrete mixtures were studied. The following results were obtained:

− The processing of TFM for 10 min leads to the grinding of not only fibers, but also granules, fragments of which are noticeable in the total mass of the substance. There is a large amount of the smallest nanosized crushed fractions, however, there is also a significant amount of larger particles of a fibrous form. This is the reason for the effective use of the material to control the structure formation of the cement composite, both at the macro level (fiber) and at the micro level (cement substitute).
− The reactive activity of TFM is confirmed by the high solubility in saturated alkaline solutions of the hydrated binder and active interaction with the Ca(OH)_2_ released as a result of the hydration of clinker minerals. These processes make it possible to purposefully synthesize low-basic calcium silicate hydrates during hydration, which are responsible for the strength properties of the finished building product.
− At 90 days of age, at 10–20% of the content of TFM, the strength indicators increase (above 40 MPa), and at 30% of the additive content, they approach the values of the control composition without additives (above 35 MPa). For all ages, the ratio of flexural and compressive strengths is at the level of 0.2, which characterizes a high reinforcing effect. The increase in strength may be associated with the pozzolanic reaction, the completeness of which increases most at a later age. In addition, the microsize of the grains of TFM waste used in this study increases the compressive strength of the mortar by acting as a compactor.
− Analysis of the results suggests the possibility of using waste milled for 10 min as an active mineral additive, as well as to give better moldability to the mixture and its micro-reinforcement to obtain fiber-reinforced concrete.

## Figures and Tables

**Table 1:** Chemical composition and specific surface area of the feedstock.

Content of Oxides, %	Mineral Wool	Microsilica	**Portland Cement**
SiO_2_	44.11	91.5	21.2
Al_2_O_3_	12.26	0.2	5.4
Fe_2_O_3_	9.44	0.7	3.2
CaO	16.00	0.4	63.8
MgO	13.19	1.5	2.0
K_2_O + Na_2_O	2.97	1.9	0.8
other	2.27	3.8	3.6
**Specific surface area, m^2^/kg**	200	22500	364

**Table 2:** Concrete mix compositions.

Mix ID	Mineral Wool Content, wt.%	Water, L/m^3^	Cement, kg/m^3^	Micro- Silica, kg/m^3^	Mineral Wool, kg/m^3^	Sand, kg/m^3^	Super- Plasticizer, %
1	0	218.2	545.5	54.5	0.0	1500	1
2	10	218.0	490.9	49.1	54.6	1498.5	1
3	20	217.8	436.3	43.6	109.2	1496.9	1
4	30	217.6	381.7	38.2	163.8	1495.4	1
5	40	217.4	327.1	32.7	225.6	1494	1

**Table 3:** Average percentage particle size distribution.

Percentile, %	Diameter, µm		
	Non-Grinded	Grinded 5 min	Grinded 10 min
10	11.05	5.55	2.78
50	91.09	50.36	14.12
90	240.94	218.77	44.53

**Table 4:** Properties of finely ground components.

Indicator	Finely Ground Mineral Components		
	Cement	Microsilica	TFM
Specific surface area by a PSH-2 device, m^2^/kg	500	1500	500
Specific surface area by Sorbi-M device (4-point BET method), m^2^/g	3.80	21.02	2.56
Pore volume with R < 19.4 nm, cm^3^/g	0.014	0.13	0.023

**Table 5:** Fresh and physico-mechanical properties of concretes.

Mix ID	Slump, cm	Slump Flow, cm	Fresh Density (ρ), kg/m^3^	∆ρ/%TFM	Density at 28 Days (ρ^28^), kg/m^3^	∆ρ^28^/%TFM
1	20	51	2341	-	2321	-
2	20	50	2334 (−0.3%)	−0.03%	2315 (−0.3%)	−0.03%
3	20	48	2326 (−0.6%)	−0.03%	2308 (−0.6%)	−0.03%
4	20	46	2320 (−0.9%)	−0.03%	2303 (−0.8%)	−0.02%
5	18	46	2320 (−0.9%)	−0.02%	2304 (−0.7%)	−0.02%

## Data Availability

Not applicable.

## References

[1] Fediuk R.S., Yushin A.M. (2015). The use of fly ash the thermal power plants in the construction. IOP Conf. Ser. Mater. Sci. Eng..

[2] Lesovik V.S. (2001). The reducing effect of argon in the plasma treatment of high-melting nonmetallic materials (a review). Glass Ceram..

[3] Jabir H.A., Abid S.R., Murali G., Ali S.H., Klyuev S., Fediuk R., Vatin N., Promakhov V., Vasilev Y. (2020). Experimental tests and reliability analysis of the cracking impact resistance of UHPFRC. Fibers.

[4] Mousavi M.A., Sadeghi-Nik A., Bahari A., Ashour A., Khayat K.H. (2022). Cement paste modified by nano-montmorillonite and carbon nanotubes. ACI Mater. J..

[5] Dabbaghi F., Sadeghi-Nik A., Libre N.A., Nasrollahpour S. (2021). Characterizing fiber reinforced concrete incorporating zeolite and metakaolin as natural pozzolans. Structures.

[6] Makul N., Fediuk R., Amran M., Zeyad A.M., Murali G., Vatin N., Klyuev S., Ozbakkaloglu T., Vasilev Y. (2021). Use of recycled concrete aggregates in production of green cement-based concrete composites: A review. Crystals.

[7] Loganina V., Sergeeva K., Fediuk R., Uvarov V., Vatin N., Vasilev Y., Amran M., Szelag M. (2021). Increase the performances of lime finishing mixes due to modification with calcium silicate hydrates. Crystals.

[8] Usanova K., Barabanshchikov Y.G. (2020). Cold-bonded fly ash aggregate concrete. Mag. Civ. Eng..

[9] Kharun M., Klyuev S., Koroteev D., Chiadighikaobi P.C., Fediuk R., Olisov A., Vatin N., Alfimova N. (2020). Heat treatment of basalt fiber reinforced expanded clay concrete with increased strength for cast-in-situ construction. Fibers.

[10] Fediuk R.S., Smoliakov A.K., Timokhin R.A., Batarshin V.O., Yevdokimova Y.G. (2018). Using thermal power plants waste for building materials. IOP Conf. Ser. Earth Environ. Sci..

[11] Loganina V.I., Frolov M.V., Skachkov Y.P. (2016). Lime composition for the walls of buildings made of aerated concrete. Proceedings of the 2016 International Symposium on Mechanical Engineering and Material Science (ISMEMS 2016).

[12] Fediuk R., Timokhin R., Mochalov A., Otsokov K., Lashina I. (2019). Performance properties of high-density impermeable cementitious paste. J. Mater. Civ. Eng..

[13] Wang B., Li K., Nan D., Feng S., Hu B., Wang T., Lu Q. (2022). Enhanced production of levoglucosenone from pretreatment assisted catalytic pyrolysis of waste paper. J. Anal. Appl. Pyrolysis.

[14] Lukuttsova N. (2015). Water films (nanofilms) in cement concrete deformations. Int. J. Appl. Eng. Res..

[15] Kolesnikov A.S. (2015). Kinetic investigations into the distillation of nonferrous metals during complex processing of waste of metallurgical industry. Russ. J. Non-Ferr. Met..

[16] Chakrawarthi V., Dharmar B., Avudaiappan S., Amran M., Flores E.S., Alam M.A., Rashid R.S. (2022). Destructive and non-destructive testing of the performance of copper slag fiber-reinforced concrete. Materials.

[17] Ibragimov R.A., Korolev E.V., Deberdeev T.R., Leksin V.V. (2019). Efficient complex activation of Portland cement through processing it in the vortex layer machine. Struct. Concr..

[18] Saidumov M.S., Murtazaeva T.-A., Salamanova M.S., Alaskhanov A.K., Ismailova Z.K., Saidumov M.S. (2019). Water-reducing and plasticizing additives for highly mobile concrete mixtures. Proceedings of the International Symposium “Engineering and Earth Sciences: Applied and Fundamental Research” Dedicated to the 85th Anniversary of H.I. Ibragimov (ISEES 2019).

[19] Amran M., Fediuk R., Murali G., Avudaiappan S., Ozbakkaloglu T., Vatin N., Karelina M., Klyuev S., Gholampour A. (2021). Fly ash-based eco-efficient concretes: A comprehensive review of the short-term properties. Materials.

[20] Fediuk R.S., Lesovik V.S., Mochalov A.V., Otsokov K.A., Lashina I.V., Timokhin R.A. (2018). Composite binders for concrete of protective structures. Mag. Civ. Eng..

[21] Ali M., Abbas S., Khan M.I., Anwar Gad M., Ammad S., Khan A. Experimental validation of Mander’s model for low strength confined concrete under axial compression. Proceedings of the 2020 Second International Sustainability and Resilience Conference: Technology and Innovation in Building Designs (51154).

[22] Zaleska M., Pavlikova M., Pavlik Z., Jankovsky O., Pokorny J., Tydlitat V., Svora P., Cerny R. (2018). Physical and chemical characterization of technogenic pozzolans for the application in blended cements. Constr. Build. Mater..

[23] Rudenko A., Biryukov A., Kerzhentsev O., Fediuk R., Vatin N., Vasilev Y., Klyuev S., Amran M., Szelag M. (2021). Nano- and Micro-modification of building reinforcing bars of various types. Crystals.

[24] Korsun V., Vatin N., Korsun A., Nemova D. Physical-mechanical properties of the modified fine-grained concrete subjected to thermal effects up to 200 °C. Proceedings of the 4th International Conference on Advanced Design and Manufacturing Engineering (ADME 2014).

[25] Tolstoy A., Lesovik V., Fediuk R., Amran M., Gunasekaran M., Vatin N., Vasilev Y. (2020). Production of greener high-strength concrete using Russian quartz sandstone mine waste aggregates. Materials.

[26] Ivanovna L.V., Vladimirovna Z.C. (2015). The effectiveness of use of the composite binder as a dry mix. Case Stud. Constr. Mater..

[27] Lukuttsova N., Pykin A., Kleymenicheva Y., Suglobov A., Efremochkin R. (2016). Nano-additives for composite building materials and their environmental safety. Int. J. Appl. Eng. Res..

[28] Kolesnikov A.S., Kenzhibaeva G.S., Botabaev N.E., Kutzhanova A.N., Iztleuov G.M., Suigenbaeva A.Z., Ashirbekov K.A., Kolesnikova O.G. (2020). Thermodynamic modeling of chemical and phase transformations in a Waelz process-slag–carbon system. Refract. Ind. Ceram..

[29] Stroganov V., Sagadeev E., Ibragimov R., Potapova L. (2020). Mechanical activation effect on the biostability of modified cement compositions. Constr. Build. Mater..

[30] Smirnova O. (2019). Compatibility of shungisite microfillers with polycarboxylate admixtures in cement compositions. ARPN J. Eng. Appl. Sci..

[31] Fediuk R., Smoliakov A., Stoyushko N. (2016). Increase in composite binder activity. IOP Conf. Ser. Mater. Sci. Eng..

[32] Lesovik V.S., Zagorodnyuk L.K., Babaev Z.K., Dzhumaniyazov Z.B. (2020). Analysis of the causes of brickwork efflorescence in the Aral Sea region. Glas. Ceram..

[33] Amran M., Murali G., Khalid N.H.A., Fediuk R., Ozbakkaloglu T., Lee Y.H., Haruna S., Lee Y.Y. (2021). Slag uses in making an ecofriendly and sustainable concrete: A review. Constr. Build. Mater..

[34] Tolstoy A.D., Lesovik V.S., Glagolev E.S., Krymova A.I. (2018). Synergetics of hardening construction systems. IOP Conf. Ser. Mater. Sci. Eng..

[35] Mukhametrakhimov R., Galautdinov A., Garafiev A. (2020). The concrete modified by conductive mineral for electrode heating. IOP Conf. Ser. Mater. Sci. Eng..

[36] Wang D., Ju Y., Shen H., Xu L. (2019). Mechanical properties of high performance concrete reinforced with basalt fiber and polypropylene fiber. Constr. Build. Mater..

[37] Raza S.S., Qureshi L.A., Ali B., Raza A., Khan M.M. (2020). Effect of different fibers (steel fibers, glass fibers, and carbon fibers) on mechanical properties of reactive powder concrete. Struct. Concr..

[38] Amiri H., Azadi S., Karimaei M., Sadeghi H., Dabbaghi F. (2022). Multi-objective optimization of coal waste recycling in concrete using response surface methodology. J. Build. Eng..

[39] Dabbaghi F., Fallahnejad H., Nasrollahpour S., Dehestani M., Yousefpour Y. (2021). Evaluation of fracture energy, toughness, brittleness, and fracture process zone properties for lightweight concrete exposed to high temperatures. Theor. Appl. Fract. Mech..

[40] Klyuyev S.V., Guryanov Y.V. (2013). External reinforcing of fiber concrete constructions by carbon fiber tapes. Mag. Civ. Eng..

[41] Mukhametrakhimov R., Lukmanova L. (2021). Investigation of Portland cement in 3D concrete printing. Lect. Notes Civ. Eng..

[42] Galautdinov A., Mukhametrakhimov R., Kupriyanov V. (2021). Gypsum-fiber radioprotective facing materials. Lect. Notes Civ. Eng..

[43] Klyuev S.V., Khezhev T.A., Pukharenko Y.V., Klyuev A.V. (2018). To the question of fiber reinforcement of concrete. Mater. Sci. Forum.

[44] Klyuev S.V., Bratanovskiy S.N., Trukhanov S.V., Manukyan H.A. (2019). Strengthening of concrete structures with composite based on carbon fiber. J. Comput. Theor. Nanosci..

[45] Fediuk R., Mugahed Amran Y.H., Mosaberpanah M.A., Danish A., El-Zeadani M., Klyuev S.V., Vatin N. (2020). A critical review on the properties and applications of sulfur-based concrete. Materials.

[46] Klyuev S.V., Khezhev T.A., Pukharenko Y.V., Klyuev A.V. (2018). Fibers and their properties for concrete reinforcement. Mater. Sci. Forum.

[47] Mukhametrakhimov R.K., Lukmanova L.V., Khuzin A.F. (2017). Investigation of the influence of organosilicon compounds on fiber cement panels’ characteristics. IOP Conf. Ser. Earth Environ. Sci..

[48] Klyuev S.V., Khezhev T.A., Pukharenko Y.V., Klyuev A.V. (2018). Fiber concrete for industrial and civil construction. Mater. Sci. Forum.

[49] Klyuyev S.V., Klyuyev A.V., Sopin D.M., Netrebenko A.V., Kazlitin S.A. (2013). Heavy loaded floors based on fine-grained fiber concrete. Mag. Civ. Eng..

[50] Klyuev S.V., Khezhev T.A., Pukharenko Y.V., Klyuev A.V. (2018). Experimental study of fiber-reinforced concrete structures. Mater. Sci. Forum.

[51] Mukhametrakhimov R.K., Galautdinov A.R., Gilmanshin I.R. (2019). Modified gypsum-cement-pozzolanic composites reinforced with polypropylene fibers. IOP Conf. Ser. Mater. Sci. Eng..

[52] Begich Y.E., Klyuev S.V., Jos V.A., Cherkashin A.V. (2020). Fine-grained concrete with various types of fibers. Mag. Civ. Eng..

[53] Lukmanova L.V., Mukhametrakhimov R.K., Gilmanshin I.R. (2019). Investigation of mechanical properties of fiber-cement board reinforced with cellulosic fibers. IOP Conf. Ser. Mater. Sci. Eng..

[54] Karmegam A., Avudaiappan S., Amran M., Guindos P., Vatin N.I., Fediuk R. (2022). Retrofitting RC beams using high-early strength alkali-activated concrete. Case Stud. Constr. Mater..

[55] Subash N., Avudaiappan S., Adish Kumar S., Amran M., Vatin N., Fediuk R., Aepuru R. (2021). Experimental investigation on geopolymer concrete with various sustainable mineral ashes. Materials.

[56] Zeyad A.M., Magbool H.M., Amran M., Mijarsh M.J.A., Almalki A. (2022). Performance of high-strength green concrete under the influence of curing methods, volcanic pumice dust, and hot weather. Arch. Civ. Mech. Eng..

[57] Amran M., Huang S.S., Onaizi A.M., Murali G., Abdelgader H.S. (2022). Fire spalling behavior of high-strength concrete: A critical review. Constr. Build. Mater..

[58] Prakash R., Divyah N., Srividhya S., Avudaiappan S., Amran M., Naidu Raman S., Fediuk R. (2022). Effect of steel fiber on the strength and flexural characteristics of coconut shell concrete partially blended with fly ash. Materials.

[59] Danish A., Mosaberpanah M.A., Salim M.U., Amran M., Fediuk R., Ozbakkaloglu T., Rashid M.F. (2022). Utilization of recycled carbon fiber reinforced polymer in cementitious composites: A critical review. J. Build. Eng..

[60] Amran M., Huang S.S., Debbarma S., Rashid R.S. (2022). Fire resistance of geopolymer concrete: A critical review. Constr. Build. Mater..

[61] Mubarak M., Muhammad Rashid R.S., Amran M., Fediuk R., Vatin N., Klyuev S. (2021). Mechanical properties of high-performance hybrid fibre-reinforced concrete at elevated temperatures. Sustainability.

[62] Lesovik V., Fediuk R., Amran M., Alaskhanov A., Volodchenko A., Murali G., Elistratkin M. (2021). 3D-printed mortars with combined steel and polypropylene fibers. Fibers.

[63] Chakrawarthi V., Avudaiappan S., Amran M., Dharmar B., Raj Jesuarulraj L., Fediuk R., Saavedra Flores E. (2021). Impact resistance of polypropylene fibre-reinforced alkali–activated copper slag concrete. Materials.

[64] Amran M., Fediuk R., Abdelgader H.S., Murali G., Ozbakkaloglu T., Lee Y.H., Lee Y.Y. (2022). Fiber-reinforced alkali-activated concrete: A review. J. Build. Eng..

[65] Amran M., Al-Fakih A., Chu S.H., Fediuk R., Haruna S., Azevedo A., Vatin N. (2021). Long-term durability properties of geopolymer concrete: An in-depth review. Case Stud. Constr. Mater..

